# Internalization of Dectin-1 terminates induction of inflammatory responses

**DOI:** 10.1002/eji.200838687

**Published:** 2009-02

**Authors:** Patricia Hernanz-Falcón, Olivier Joffre, David L Williams, Caetano Reis e Sousa

**Affiliations:** 1 Immunobiology Laboratory, Cancer Research UK, London Research Institute, Lincoln's Inn Fields LaboratoriesLondon, UK; 2 Department of Surgery, James H. Quillen College of MedicineJohnston City, TN, USA

**Keywords:** DC, Dectin-1, Phagocytosis, Syk

## Abstract

Dectin-1 is a pattern-recognition receptor recognizing β-(1,3)-glucans found on fungal cell walls. Dectin-1 plays an important role in immunity to fungi by mediating phagocytic clearance of fungal particles and inducing transcription of innate response genes. We show here that the two processes are linked and that Dectin-1 signalling for inflammation is attenuated by phagocytosis. Blocking Dectin-1 ligand-dependent internalization using either actin polymerization or dynamin inhibitors, large non-phagocytosable β-glucan particles or poorly phagocytic cells leads in all cases to enhanced and sustained activation of downstream signalling pathways and culminates in production of high levels of pro-inflammatory cytokines. These findings establish the importance of phagocytosis not only in the clearance of pathogens, but also in the modulation of pattern-recognition receptor signalling and strongly suggest that internalization is the first step to attenuation of Dectin-1-mediated pro-inflammatory responses.

## Introduction

Direct recognition of microbes by pattern-recognition receptors on DC and MΦ is central to initiating immune responses against infectious organisms 1. TLR are most prominent among the pattern-recognition receptors involved in pro-inflammatory processes, but we and others have recently shown that Dectin-1 is a non-TLR receptor that also has the capacity to induce the expression of innate response genes in DC and MΦ 2–8. Dectin-1 is a C-type lectin expressed on myeloid cells that binds to β-1,3-glucans found on the cell walls of fungi and some bacteria 2. Dectin-1 signals *via* a novel hemITAM motif that becomes phosphorylated by Src family kinases on receptor engagement 9, 10. This allows recruitment and activation of the spleen tyrosine kinase (Syk), which then couples to downstream pathways, including those leading to production of reactive oxygen species and, *via* the adaptor CARD9, to the activation of NF-κB 7, 9–11. The latter, together with NFAT and transcription factors activated by MAP kinases downstream of Syk, regulates the expression of a plethora of innate response genes, including those encoding co-stimulatory molecules and pro-inflammatory cytokines and chemokines 7, 9–12. Notably, unlike the TLR, Dectin-1 not only regulates gene expression but can also function as a phagocytic receptor 10, 13, 14. Phagocytosis is one of the first lines of defense from infection and is a complex process involving mobilization of the actin cytoskeleton and subsequent particle engulfment 14, 15. To what extent Dectin-1 internalization during phagocytosis impacts on signalling for innate gene induction remains unclear. We show here that blocking of Dectin-1 ligand-dependent internalization leads to sustained activation of MAP kinases and increased cytokine production. These results demonstrate the importance of phagocytosis not only in the clearance of the pathogen but also in the modulation of C-type lectin-initiated inflammatory responses.

## Results and discussion

### β-glucan microparticles are weak inducers of Dectin-1-dependent cytokines

Different particulate β-glucans have been used to study signalling by Dectin-1 in MΦ and DC. Some investigators, including ourselves, have previously utilized curdlan, a large particulate (1,3)-β-glucan from *Alcaligenes faecalis* 16, to demonstrate that triggering of the Dectin-1/Syk pathway independently of TLR signalling promotes DC activation, including secretion of cytokines such as IL-6, TNF-*α*, IL-2, IL-10 and IL-12p40 7, 17. Others have used β-glucan microparticles (Glu-mp), a highly purified microparticulate form of (1,3)-β-d-glucan from *Saccharomyces cerevisiae*, to stimulate MΦ 3, 18, 19. In those cells, Glu-mp induces phosphorylation of both Syk kinase and its substrate SLP-76 but does not lead to cytokine and chemokine production 3. However, Glu-mp potently synergises with TLR agonists in promoting the activation of NF-κB and the production of TNF-α, MIP-1*α* and MIP-2 3. These studies suggested that distinct types of β-glucan vary in their ability to trigger Dectin-1-dependent innate responses and/or that DC and MΦ possess intrinsically distinct responses to Dectin-1 engagement.

**Figure 1:**
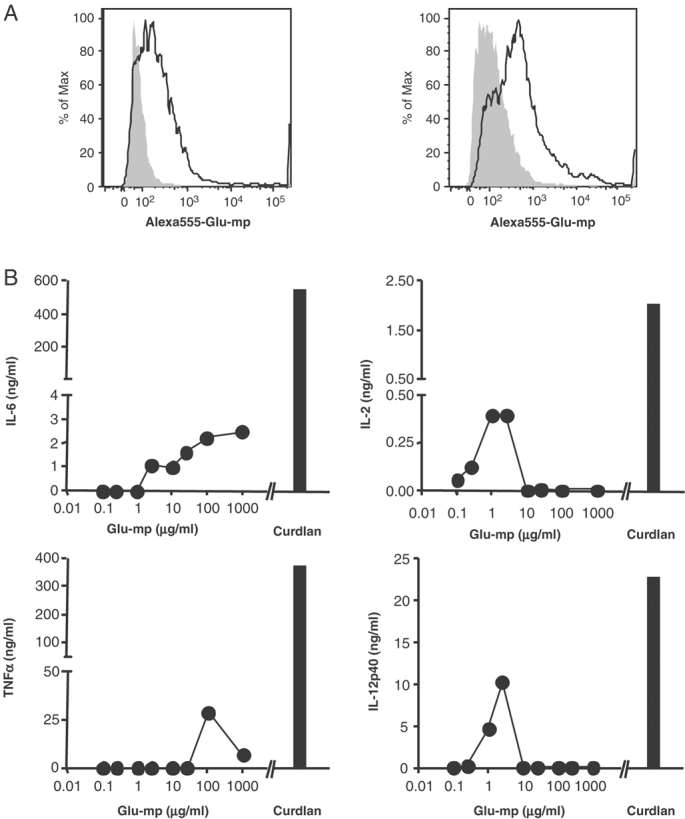
Glu-mp is weaker than curdlan at inducing inflammatory cytokine production. (A) HEK293 cells expressing Dectin-1 (black line, left panel) or C57BL/6 BMDC (black line, right panel) bind Alexa555-labelled Glu-mp, as shown by flow cytometric analysis. Binding is not observed in untransfected HEK293 cells (gray, left panel) or Dectin-1-deficient BMDC (gray, right panel). One representative out of three experiments is shown. (B) Production of IL-6, TNF-α, IL-2 and IL-12p40 by C57BL/6 BMDC stimulated with the indicated doses of Glu-mp or with 100 μg/mL curdlan. Cytokine concentration in 16 h culture supernatants was determined by ELISA. Data shown are mean of duplicate wells. One representative out of three experiments is shown.

To test the former hypothesis, we compared the ability of Glu-mp and curdlan with activate DC, using production of inflammatory cytokines as the major readout. First, we confirmed that, like curdlan 7, Glu-mp preparations acted as specific ligands for Dectin-1. We found that fluorescent Glu-mp binds to Dectin-1-transfected HEK293 cells but not to the parental cell line (Fig. 1A, left). In addition, fluorescent Glu-mp bound to WT bone-marrow-derived DC (BMDC) but not to cells derived from Dectin-1-deficient mice, indicating that DC do not possess compensatory receptors for binding β-glucans (Fig. 1A, right). To directly compare the ability of curdlan and Glu-mp preparations to stimulate DC, cells were cultured with curdlan or Glu-mp and the levels of IL-6, IL-2, TNF-α and IL-12p40 in supernatants were measured after 16 h. Glu-mp induced much lower levels of cytokines when compared with curdlan (Fig. 1B). Extensive titration studies established that even when used at maximal dose, Glu-mp induced at best one-hundredth to one-tenth the amount of TNF-α and IL-6 that could be elicited by an optimal dose of curdlan (Fig. 1B). Thus, Glu-mp is markedly weaker than curdlan at stimulating DC. This is consistent with the data obtained with MΦ 3 and therefore suggests that the differences previously noted for Glu-mp and curdlan are not only attributable to the use of different cell types 3, 7.

### Blocking phagocytosis converts Glu-mp into a potent activator of innate responses *via* the Dectin-1 pathway

**Figure 2:**
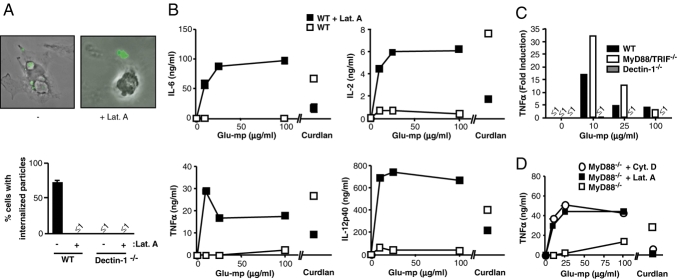
Block of phagocytosis converts Glu-mp into a potent DC activator. (A) C57BL/6 or Dectin-1-deficient BMDC were treated with or without latrunculin A for 30 min prior to addition of Alexa647-labelled Glu-mp. After 1 h incubation, cells were washed, fixed and visualized by confocal microscopy. Data are the mean particle uptake and SD of three independent experiments. (B) C57BL/6 BMDC were stimulated with different doses of Glu-mp or with 100 μg/mL curdlan in the presence or in the absence of latrunculin A. IL-6, TNF-α, IL-2 and IL-12p40 in culture supernatants were determined by ELISA. Data shown are mean of duplicate wells. One representative out of three independent experiments is shown. (C) TNF-α concentrations were determined by ELISA in the supernatant of C57BL/6, MyD88/TRIF doubly deficient, or Dectin-1-deficient BMDC stimulated with the indicated amounts of Glu-mp in the presence or absence of latrunculin A. Data are represented as fold TNF-α induction, dividing the value obtained with latrunculin A-treated cells by that obtained with untreated cells (where no induction was observed, data are shown as ≤1). Data shown are mean of duplicate wells. One representative out of three independent experiments is shown. (D) MyD88-deficient BMDC were stimulated with different doses of Glu-mp or with 100 μg/mL curdlan in the presence or in the absence of latrunculin A or cytochalasin D. TNF-α concentrations in culture supernatants were determined by ELISA. Data shown are mean of duplicate wells. One representative out of three independent experiments is shown.

Curdlan forms insoluble particles of >100 μm diameter that cannot be phagocytosed (data not shown). In contrast, Glu-mp size is approximately 1 μm and, even though the particles have a tendency to form larger aggregates (5 μm), they are readily phagocytosed by either MΦ or DC (Fig. 2A). To examine if internalization of Glu-mp affects its ability to stimulate DC activation, we blocked phagocytosis using actin polymerization inhibitors 20. Like binding, the uptake of Glu-mp was Dectin-1-dependent and was completely blocked by pre-treatment of DC with latrunculin A (Fig. 2A). Notably, such blockade converted Glu-mp into a potent stimulus for induction of IL-6, IL-2, TNF-α and IL-12p40 (Fig. 2B). The effect was not restricted to latrunculin A as it could be mimicked with other actin polymerization inhibitors such as cytochalasin D, all of which rendered Glu-mp as potent a stimulus as curdlan (Fig. 2B and D, and data not shown). Interestingly, the potency of the latter was paradoxically reduced by DC treatment with the same drugs (Fig. 2B), possibly because actin inhibition impairs the ability of DC to migrate and form conjugates with the large curdlan particles (data not shown). The potentiation of the Glu-mp stimulatory activity by actin polymerization inhibitors was not due to the presence of contaminating TLR agonists in the drug preparations, which acted in a synergistic fashion with Dectin-1 signals 3, 4, 8, because it was still observed when using MyD88-deficient or MyD88/TRIF doubly deficient BMDC that lack all TLR signalling (Fig. 2C and D). In addition, the stimulatory activity of Glu-mp in the presence of the drugs remained strictly Dectin-1-dependent (Fig. 2C), as expected. In contrast, the response to a control TLR9 agonist such as CpG was Dectin-1-independent and MyD88/TRIF-dependent (data not shown). These data indicate that manipulation of actin-dependent endocytic processes markedly affects the ability of Dectin-1 agonists to induce pro-inflammatory responses. In this regard, the previously noted potency of curdlan as an innate stimulus appears to relate to its ability to form large particles that cannot be internalized.

### Block of Glu-mp phagocytosis correlates with sustained signalling by Dectin-1 and MAPK activation

**Figure 3:**
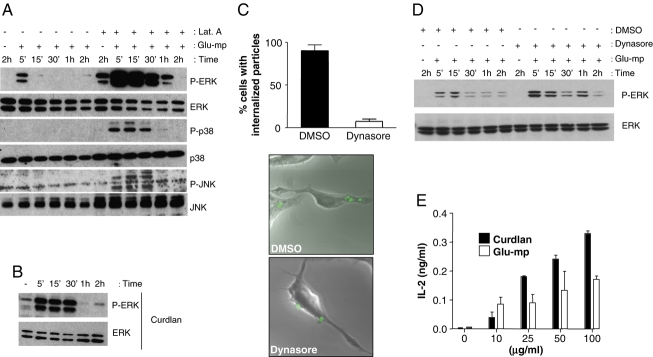
Block of Glu-mp phagocytosis promotes sustained MAPK activation. (A) C57BL/6 BMDC were stimulated with or without 100 μg/mL Glu-mp in the presence or absence of latrunculin A, as indicated. Activation of p38, ERK and JNK was analyzed by immunoblotting with antibodies against the phosphorylated form of the kinases. The blots were re-probed with antibodies against total kinase as a loading control. One representative out of four independent experiments is shown. (B) The same experiment conducted with or without (−) 100 μg/mL curdlan. Activation of ERK was analyzed by immunoblotting with antibodies against the phosphorylated form of the kinase. One representative out of four independent experiments is shown. (C) HEK293 cells expressing Dectin-1 were treated with DMSO or with dynasore for 30 min prior to addition of Alexa647-labelled Glu-mp (50 μg/mL). After 1 h incubation, cells were washed, fixed and visualized by confocal microscopy. Data are the mean particle uptake and SD of three independent experiments. (D) HEK293 cells expressing Dectin-1 were stimulated with 50 μg/mL Glu-mp or without stimulus for the indicated periods of time, in the presence of DMSO or dynasore as indicated. Activation of ERK was analyzed by immunoblotting with antibodies against the phosphorylated form of the kinase. The blots were re-probed with antibodies against total kinase as a loading control. One representative out of three independent experiments is shown. (E) LK cells expressing Dectin-1 were stimulated with different doses of curdlan or Glu-mp. IL-2 concentrations in culture supernatants were determined by ELISA. Data shown are mean±SD of triplicate wells. One representative out of three independent experiments is shown.

Internalization of cell surface signalling receptors can result in two distinct outcomes. Some receptors, such as TGF-β receptor, nerve growth factor receptor and epidermal growth factor receptor continue to signal from endosomes, amplifying downstream responses before being eventually degraded 21–26. In contrast, for the BCR, uptake is associated with diminished Ca2^+^ fluxes and decreased activation of RelA, Akt and ERK, suggesting that productive signalling occurs before receptor translocation to endosomes 27–30. To determine the effect of internalization on Dectin-1 signalling, we evaluated MAP kinase (ERK, p38, JNK) activation in BMDC stimulated with Glu-mp in the presence or absence of actin polymerization inhibitors. Cells stimulated with Glu-mp in the absence of latrunculin A displayed weak and transient phosphorylation of ERK and no detectable activation of p38 or JNK (Fig. 3A). In contrast, in cells stimulated in the presence of latrunculin A, we observed a strong and sustained activation of all three MAP kinases (Fig. 3A), mimicking the action of curdlan (Fig. 3B and Ref. 7).

To prove that the actual lack of internalization rather than a general actin polymerization block is responsible for enhancing Dectin-1-mediated inflammatory responses, we analyzed the effect of a dynamin inhibitor, dynasore 31–33. Because this reagent needs to be used in serum-free conditions, which are not compatible with BMDC survival (data not shown), we utilized HEK293 cells stably transfected with Dectin-1. Like DC, such transfectants avidly internalized Glu-mp and this was fully blocked in the presence of dynasore (Fig. 3C). Notably, the same cells stimulated with Glu-mp in the presence of dynasore showed strong and sustained phosphorylation of ERK (Fig. 3D). In contrast to latrunculin A (Fig. 3A), dynasore did not induce ERK phosphorylation by itself (Fig. 3D), supporting the notion that the strong MAPK activation observed is selectively attributable to blockade of internalization.

Finally, to diminish particle internalization while avoiding possible non-specific effects associated with chemical inhibitors, we tested cells with low phagocytic activity. In contrast to Dectin-1-expressing HEK293 cells, the B-cell hybridoma LK35.2 does not avidly internalize Glu-mp even after transfection with Dectin-1 (data not shown). We therefore stimulated LK cells expressing Dectin-1 with Glu-mp and curdlan at different doses and evaluated their ability to produce IL-2 as a readout of Dectin-1/Syk activation 9. Notably, in contrast to its low stimulatory activity on DC, Glu-mp acted as a significant inducer of cytokine production by Dectin-1-expressing LK cells, being only slightly weaker than curdlan (Fig. 3E). Thus, in poorly phagocytic cells, curdlan and Glu-mp act as comparable agonists for Dectin-1-mediated inflammatory responses.

## Concluding remarks

In this study, we have used two specific ligands, curdlan and Glu-mp, to explore the effect of Dectin-1 internalization on downstream responses. We show here that curdlan is much more potent than Glu-mp at stimulating pro-inflammatory gene expression in DC and that this correlates with the inability of the cells to ingest curdlan particles. Notably, we show that Glu-mp can be converted into a “curdlan-like” stimulus simply by blocking phagocytic uptake, using dynamin or actin polymerization inhibitors, or by using poorly phagocytic cells unable to efficiently internalize Glu-mp. Notably, during the revision of this paper, complementary results were reported by Rosas *et al*., who similarly conclude that Dectin-1-mediated pro-inflammatory responses are associated with “frustrated phagocytosis” of β-glucan-containing particles 34. Taken together, the results from the two studies suggest that internalization of Dectin-1 following interaction with ligand leads to attenuation of signalling pathways involved in innate gene induction. This does not exclude the possibility that Dectin-1 may still signal inside the endosome for alternative responses like reactive oxygen species production 10, 13. Nevertheless, our data suggest that Dectin-1 behaves like another Syk-coupled receptor, the BCR, in that it signals primarily from the cell surface for induction of gene expression 27–30. The explanation for this phenomenon remains unclear even in the case of the BCR but it could be that phosphatases involved in attenuation of the signal have better access to Dectin-1 once the receptor is internalized or that the assembly or stability of signalling complexes is physically disrupted by receptor endocytosis 27–30. Our observations on the regulation of Dectin-1 signalling may therefore be particularly relevant for understanding the role of innate immune receptors such as C-type lectins that play a dual role in microbe phagocytosis and in inducing inflammation.

## Materials and methods

### Mice

C57BL/6 mice, MyD88-deficient mice (a gift from Shizuo Akira, Osaka University, Osaka, Japan), and TRIF×MyD88 doubly deficient mice were bred at CRUK. Bone marrow cells from Dectin-1-deficient mice 35 were a gift from Gordon Brown (University of Cape Town, South Africa). All animal experiments were performed in accordance with national and institutional guidelines for animal care.

### Reagents

Culture medium was RPMI 1640 (Invitrogen) supplemented with glutamine, penicillin, streptomycin, 2-mercaptoethanol, non-essential amino acids, sodium pyruvate, Hepes (all from Invitrogen) and 10% heat-inactivated fetal bovine serum (Bioclear). Curdlan was obtained from Wako and suspended in PBS at 10 mg/mL. CpG oligonucleotide 1668 was synthesized by Sigma. GM-CSF was made by Cancer Research UK protein purification service and batches were titrated to give optimal growth conditions for BMDC. Glu-mp and Alexa 647/555-labelled Glu-mp were generated as described 18, 36.

Antibodies used for Western blotting were purchased from Cell Signaling.

Cytochalasin D and latruculin A were purchased from Calbiochem. Dynasore was purchased from Sigma.

### Flow cytometry

Cell suspensions were incubated with Alexa555-labelled Glu-mp in complete RPMI at 37°C for 30 min, washed twice with PBS, fixed with Fix and Perm Reagent A (Caltag Laboratories, Burlingame, CA, USA), and then resuspended in ice-cold PBS supplemented with 2 mM EDTA, 1% FBS and 0.02% sodium azide. Data were acquired on a FACSCalibur (BD Biosciences) and analyzed using FlowJo software (Treestar, San Carlos, CA, USA).

### BMDC culture and stimulation

BMDC were generated using GM-CSF as previously described 37 and were purified from bulk cultures with anti-CD11c microbeads (Miltenyi Biotec). BMDC purity was checked by FACS and was routinely higher than 95%. For cytokine production analyses, 5–10×10^4^ BMDC were cultured in each well of a 96-well round-bottomed plate, for 18–24 h, in 200 μL culture medium supplemented with GM-CSF in the presence of 100 μg/mL curdlan, 500 ng/mL CpG or indicated amount of Glu-mp. Cytokine levels were measured in the supernatants by sandwich ELISA. Actin polymerization inhibitors were added 30 min prior stimulation with the ligands and maintained throughout the experiment (2.5 μM).

### Cell lines culture and stimulation

HEK293 cells expressing Dectin-1 were cultured for 24 h in serum-free DMEM (Invitrogen) supplemented with glutamine, penicillin, streptomycin (all from Invitrogen). Dynasore was re-suspended in DMSO, added 30 min prior stimulation with Glu-mp (50 μg/mL), and maintained throughout the experiment.

LK cells expressing Dectin-1 9, 10 were cultured in RPMI 1640 supplemented with glutamine, penicillin, streptomycin, 2-mercaptoethanol and 10% heat-inactivated FBS. The cells were stimulated with Glu-mp (50 μg/mL) or curdlan (50 μg/mL).

### Confocal microscopy

C57BL/6 or Dectin-1-deficient BMDC, HEK293 cells expressing Dectin-1 and LK cells expressing Dectin-1 were allowed to adhere to fibronectin-coated dishes (MatTek corporation). Alexa647-labelled Glu-mp or FITC-labelled zymosan was added at 100 μg/mL *per* well and the cultures were incubated at 37°C for 1 h. Cells were fixed with Fix and Perm Reagent A (Caltag Laboratories) and then mounted with Fluoromount-G (SouthernBiotech).

A confocal series of differential interference contrast and fluorescence images was obtained simultaneously with a laser scanning confocal microscope (Axioplan 2, Zeiss, Germany) with a 63°-NA 1.4 oil objective. Image analysis was performed with LSM 510 software (Zeiss, Germany). For the quantification of internalization, 300 cells were counted (100 cells *per* field) in each experiment and data shown are representative of three independent experiments.

### Western blotting

Cells were harvested by scraping in ice-cold PBS supplemented with 5 mM EDTA. After centrifugation, the cell pellet was lysed with RIPA buffer (50 mM Tris, pH 7.5, 1% NP-40, 0.5% deoxycholic acid, 0.1% SDS, 150 mM NaCl, 10 mM NaF, 1 mM Na_3_VO_4_, 2 mM Na_4_P_2_O_7_ plus a mixture of protease inhibitors (Roche Molecular Biochemicals)) for 30 min on ice. Cell debris were removed by centrifugation and quantitated by protein assay (Bio Rad). For Western blotting, a fixed amount of total protein was mixed with sample buffer (0.125 M Tris, pH 6.8, 4% SDS, 20% glycerol, 5% 2-mercaptoethanol), and resolved by 4–20% acrylamide gradient Tris-glycine SDS-PAGE (Invitrogen). After transferring to PDVF membrane (Millipore), proteins were analyzed by immunoblotting and visualized by ECL (Pierce).

## References

[1] Janeway CA (1989). Approaching the asymptote? Evolution and revolution in immunology. Cold Spring Harb. Symp. Quant. Biol..

[2] Brown GD, Gordon S (2001). Immune recognition. A new receptor for beta-glucans. Nature.

[3] Dennehy KM, Ferwerda G, Faro-Trindade I, Pyz E, Willment JA, Taylor PR, Kerrigan A (2008). Syk kinase is required for collaborative cytokine production induced through Dectin-1 and Toll-like receptors. Eur. J. Immunol..

[4] Gantner BN, Simmons RM, Canavera SJ, Akira S, Underhill DM (2003). Collaborative induction of inflammatory responses by dectin-1 and Toll-like receptor 2. J. Exp. Med..

[5] Dennehy KM, Brown GD (2007). The role of the beta-glucan receptor Dectin-1 in control of fungal infection. J. Leukoc. Biol..

[6] Slack EC, Robinson MJ, Hernanz-Falcón P, Brown GD, Williams DL, Schweighoffer E, Tybulewicz VL, Reis e Sousa C (2007). Syk-dependent ERK activation regulates IL-2 and IL-10 production by DC stimulated with zymosan. Eur. J. Immunol..

[7] LeibundGut-Landmann S, Gross O, Robinson MJ, Osorio F, Slack EC, Tsoni SV, Schweighoffer E (2007). Syk- and CARD9-dependent coupling of innate immunity to the induction of T helper cells that produce interleukin 17. Nat. Immunol..

[8] Brown GD, Herre J, Williams DL, Willment JA, Marshall AS, Gordon S (2003). Dectin-1 mediates the biological effects of beta-glucans. J. Exp. Med..

[9] Rogers NC, Slack EC, Edwards AD, Nolte MA, Schulz O, Schweighoffer E, Williams DL (2005). Syk-dependent cytokine induction by Dectin-1 reveals a novel pattern recognition pathway for C type lectins. Immunity.

[10] Underhill DM, Rossnagle E, Lowell CA, Simmons RM (2005). Dectin-1 activates Syk tyrosine kinase in a dynamic subset of macrophages for reactive oxygen production. Blood.

[11] Gross O, Gewies A, Finger K, Schafer M, Sparwasser T, Peschel C, Forster I, Ruland J (2006). Card9 controls a non-TLR signalling pathway for innate anti-fungal immunity. Nature.

[12] Goodridge HS, Simmons RM, Underhill DM (2007). Dectin-1 stimulation by *Candida albicans* yeast or zymosan triggers NFAT activation in macrophages and dendritic cells. J. Immunol..

[13] Gantner BN, Simmons RM, Underhill DM (2005). Dectin-1 mediates macrophage recognition of *Candida albicans* yeast but not filaments. EMBO J..

[14] Herre J, Marshall AS, Caron E, Edwards AD, Williams DL, Schweighoffer E, Tybulewicz V (2004). Dectin-1 uses novel mechanisms for yeast phagocytosis in macrophages. Blood.

[15] Aderem A, Underhill DM (1999). Mechanisms of phagocytosis in macrophages. Annu. Rev. Immunol..

[16] Harada T, Misaki A, Saito H (1968). Curdlan: a bacterial gel-forming beta-1,3-glucan. Arch. Biochem. Biophys..

[17] Yoshitomi H, Sakaguchi N, Kobayashi K, Brown GD, Tagami T, Sakihama T, Hirota K (2005). A role for fungal beta-glucans and their receptor Dectin-1 in the induction of autoimmune arthritis in genetically susceptible mice. J. Exp. Med..

[18] Ensley HE, Tobias B, Pretus HA, McNamee RB, Jones EL, Browder IW, Williams DL (1994). NMR spectral analysis of a water-insoluble (1-3)-beta-d-glucan isolated from *Saccharomyces cerevisiae*. Carbohydr. Res..

[19] Suram S, Brown GD, Ghosh M, Gordon S, Loper R, Taylor PR, Akira S (2006). Regulation of cytosolic phospholipase A2 activation and cyclooxygenase 2 expression in macrophages by the beta-glucan receptor. J. Biol. Chem..

[20] de Oliveira CA, Mantovani B (1988). Latrunculin A is a potent inhibitor of phagocytosis by macrophages. Life Sci..

[21] Grimes ML, Zhou J, Beattie EC, Yuen EC, Hall DE, Valletta JS, Topp KS (1996). Endocytosis of activated TrkA: evidence that nerve growth factor induces formation of signaling endosomes. J. Neurosci..

[22] Vieira AV, Lamaze C, Schmid SL (1996). Control of EGF receptor signaling by clathrin-mediated endocytosis. Science.

[23] Howe CL, Valletta JS, Rusnak AS, Mobley WC (2001). NGF signaling from clathrin-coated vesicles: evidence that signaling endosomes serve as a platform for the Ras-MAPK pathway. Neuron.

[24] Hayes S, Chawla A, Corvera S (2002). TGF beta receptor internalization into EEA1-enriched early endosomes: role in signaling to Smad2. J. Cell Biol..

[25] Wang Y, Pennock S, Chen X, Wang Z (2002). Endosomal signaling of epidermal growth factor receptor stimulates signal transduction pathways leading to cell survival. Mol. Cell. Biol..

[26] Miaczynska M, Christoforidis S, Giner A, Shevchenko A, Uttenweiler-Joseph S, Habermann B, Wilm M (2004). APPL proteins link Rab5 to nuclear signal transduction via an endosomal compartment. Cell.

[27] Stoddart A, Jackson AP, Brodsky FM (2005). Plasticity of B cell receptor internalization upon conditional depletion of clathrin. Mol. Biol. Cell.

[28] Blery M, Tze L, Miosge LA, Jun JE, Goodnow CC (2006). Essential role of membrane cholesterol in accelerated BCR internalization and uncoupling from NF-kappa B in B cell clonal anergy. J. Exp. Med..

[29] Gazumyan A, Reichlin A, Nussenzweig MC (2006). Ig beta tyrosine residues contribute to the control of B cell receptor signaling by regulating receptor internalization. J. Exp. Med..

[30] Jacob M, Todd L, Sampson MF, Pure E (2008). Dual role of Cbl links critical events in BCR endocytosis. Int. Immunol..

[31] Thompson HM, McNiven MA (2006). Discovery of a new ‘dynasore’. Nat. Chem. Biol..

[32] Macia E, Ehrlich M, Massol R, Boucrot E, Brunner C, Kirchhausen T (2006). Dynasore, a cell-permeable inhibitor of dynamin. Dev. Cell.

[33] Kagan JC, Su T, Horng T, Chow A, Akira S, Medzhitov R (2008). TRAM couples endocytosis of Toll-like receptor 4 to the induction of interferon-beta. Nat. Immunol..

[34] Rosas M, Liddiard K, Kimberg M, Faro-Trindade I, McDonald JU, Williams DL, Brown GD, Taylor PR (2008). The induction of inflammation by dectin-1 in vivo is dependent on myeloid cell programming and the progression of phagocytosis. J. Immunol..

[35] Taylor PR, Tsoni SV, Willment JA, Dennehy KM, Rosas M, Findon H, Haynes K (2007). Dectin-1 is required for beta-glucan recognition and control of fungal infection. Nat. Immunol..

[36] Ozment-Skelton TR, Goldman MP, Gordon S, Brown GD, Williams DL (2006). Prolonged reduction of leukocyte membrane-associated Dectin-1 levels following beta-glucan administration. J. Pharmacol. Exp. Ther..

[37] Inaba K, Inaba M, Romani N, Aya H, Deguchi M, Ikehara S, Muramatsu S, Steinman RM (1992). Generation of large numbers of dendritic cells from mouse bone marrow cultures supplemented with granulocyte/macrophage colony-stimulating factor. J. Exp. Med..

